# Atezolizumab-Induced Ulcerative Colitis in Patient with Hepatocellular Carcinoma: Case Report and Literature Review

**DOI:** 10.3390/medicina60091422

**Published:** 2024-08-30

**Authors:** Hyuk Kim, Yoon E Shin, Hye-Jin Yoo, Jae-Young Kim, Jeong-Ju Yoo, Sang Gyune Kim, Young Seok Kim

**Affiliations:** 1 Department of Internal Medicine, Soonchunhyang University Bucheon Hospital, Bucheon 14584, Republic of Korea; qwester123457@gmail.com (H.K.); passion_97@naver.com (Y.E.S.); 2 Department of Internal Medicine, Soonchunhyang University School of Medicine, Cheonan 31151, Republic of Korea; 117688@schmc.ac.kr (H.-J.Y.); researchfactory.kjy@gmail.com (J.-Y.K.); 3 Division of Gastroenterology and Hepatology, Department of Internal Medicine, Soonchunhyang University Bucheon Hospital, Bucheon 14584, Republic of Korea; mcnulty@schmc.ac.kr (S.G.K.); liverkys@schmc.ac.kr (Y.S.K.)

**Keywords:** immune checkpoint inhibitor colitis, UC-mimicking colitis, atezolizumab adverse effect

## Abstract

*Background and Objectives:* Immune check inhibitor (ICI) colitis is one of most common and adverse side effects of ICI. However, there was no case report of ulcerative colitis (UC)-mimicking colitis after atezolizumab use in hepatocellular carcinoma (HCC) to our knowledge. *Materials and Methods:* We would like to introduce the case of a patient with Stage IV HCC who complained of abdominal pain, diarrhea and rectal bleeding after two cycles of atezolizumab/bevacizumab chemotherapy and was then diagnosed with UC-mimicking colitis. *Results:* Endoscopy revealed typical findings of UC, suggesting diagnosis of UC-mimicking colitis. The patient was treated with systemic steroids and oral mesalamine, which significantly improved his symptoms, which were also supported by endoscopic findings. The patient resumed chemotherapy with atezolizumab and bevacizumab without any interruption to the chemotherapy schedule. *Conclusions:* Early endoscopic evaluation is pivotal to diagnosing UC-mimicking colitis. If diagnosed, UC-based treatments such as steroids and mesalamine should be strongly considered. Given previous reports of inflammatory bowel disease (IBD) flare-ups after immunotherapy, routine lower endoscopy, performed together with upper endoscopy before atezolizumab/bevacizumab therapy, is promising to patients.

## 1. Introduction

Immunotherapy using immune checkpoint inhibitor (ICI) is a promising treatment in cancer therapy [1,2], and the combination therapy using atezolizumab and bevacizumab has become standard regimen for hepatocellular carcinoma (HCC) Barcelona Clinic Liver cancer (BCLC) stage C patients [3,4]. Atezolizumab, an anti-programmed cell death-ligand 1 (PD-L1) antibody, is a widely used ICI that is part of standard therapy in non-small-cell lung cancer (NSCLC), small-cell lung cancer (SCLC) and HCC, and second-line therapy in other cancerous diseases. Bevacizumab, an anti-vascular endothelial growth factor (VEGF) antibody, is first-line therapy in metastatic colorectal cancer, metastatic cervical cancer, HCC and also used in non-cancerous angiogenic disorders such as hereditary hemorrhagic telangiectasia. Immunotherapy is considered less noxious than conventional cytotoxic chemotherapy [1]. However, there are still blind spots such as colitis, dermatitis, hepatitis, pneumonitis and bleeding that sometimes lead to death [5,6,7]. Among those, ICI colitis is the second frequent immune-related adverse event (IrAE) following dermatologic IrAEs and is quite frustrating [8]. The overall incidence of ICI colitis is reported to be ranging from 1 to 25 percent, which varies based on the agents used [9].

Atezolizumab/bevacizumab combination therapy more frequently causes colitis than sorafenib, a multiple anti-VEGF and VEGF receptor antibody in HCC. In the IMBRAVE 150 trial, a landmark trial after which atezolizumab/bevacizumab combination therapy became the standard regimen of HCC BCLC stage C patients, the incidence of colitis was three times higher in the atezolizumab/bevacizumab combination therapy group than the sorafenib group [3].

There are wide range of manifestations and severity of ICI colitis, from mild diarrhea to even refractory severe abdominal pain, and hematochezia, which leads up to surgical intervention. However, these symptoms can appear in patients with ulcerative colitis. To differentiate between them, endoscopic findings are crucial, and they sometimes reveal ulcerative colitis (UC)-like features. In cases with endoscopic findings similar to those of UC and symptom onset being compatible with a diagnosis of ICI colitis, UC-mimicking ICI colitis is diagnosed.

There are already multiple cases of ICI colitis related to various ICIs; however, only few have shown UC-mimicking features, and all were associated with ICIs other than atezolizumab [10,11,12,13,14]. And to the best of our knowledge, there were only a few case reports of severe colitis related with atezolizumab/bevacizumab combination therapy in HCC, and none of them showed UC-mimicking features. Here, we report the first case of UC-mimicking colitis induced by atezolizumab, which was responsive to steroid and mesalamine treatment.

## 2. Case Presentation

**Figure 1:**
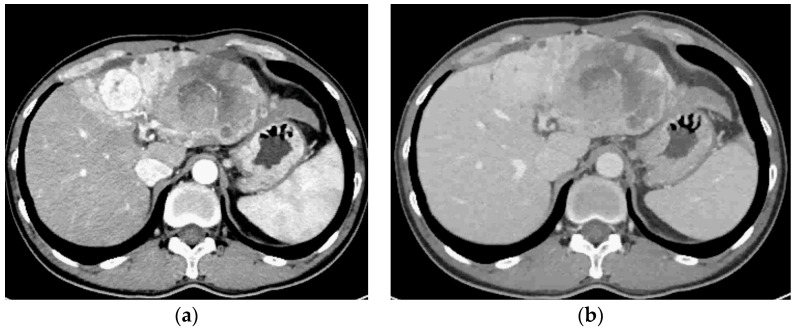
Pre-treatment liver dynamic CT with enhancement at diagnosis of HCC: (**a**) arterial phase; (**b**) portal phase.

**Figure 2:**
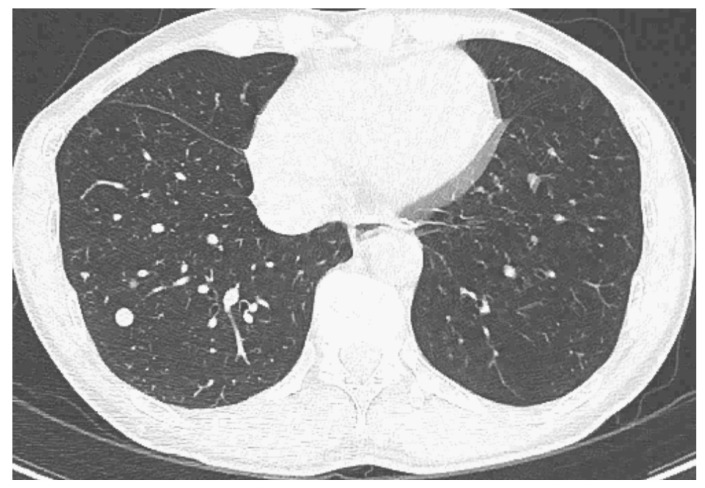
Pre-treatment chest CT with enhancement, showing multiple lung metastases.

A 56-year-old South Korean man initially visited the local clinic complaining of abdominal discomfort. His liver mass was observed on abdominal ultrasound, and after, he was referred to our hospital, a tertiary hospital in South Korea. Vital signs were stable, and the results of a physical exam were nonspecific. Laboratory findings were as follows: a white blood cell count of 5.27 × 10^3^/μL; hemoglobins, 14.1 g/dL; platelets, 191 × 10^3^/μL; prothrombin time/international normalized ratio, 1.08; total bilirubin, 0.57 mg/dL; and albumin, 3.6 g/dL. Hepatitis B surface antigen was positive, hepatitis B e antigens was positive, and the HBV DNA level was 119,234 IU/mL. The patient’s alpha-fetoprotein (AFP) was 18.5 ng/mL, and protein induced by vitamin K absence or antagonist-II (PIVKA-II) was elevated to 8687.4 mAU/mL. Three-phase computed tomography (CT) revealed an 8.3-sized mass with arterial phase enhancement and portal phase washout (Figure 1). Chest CT showed multiple lung metastases (Figure 2).

Hence, the patient was diagnosed with advanced HCC, Barcelona Clinic Liver Cancer (BCLC) stage C and modified Union for International Cancer Control (mUICC) Stage IVB, which invaded nearly the entirety of the left lobe and metastasized to regional lymph nodes and lungs. The patient started taking tenofovir disoproxil fumarate for the chronic hepatitis B. The Eastern Cooperative Oncology Group (ECOG) performance status of the patient was 0, with Child-Pugh class A (score of 5) and Model for End-stage Liver Disease (MELD) score of 7, and we decided to start atezolizumab/bevacizumab treatment for advanced HCC with lung metastasis.

There was no symptom related to ICI during the first cycle of atezolizumab/bevacizumab or the very day of the second cycle. However, on the day after the second cycle, the patient suddenly experienced abdominal pain and diarrhea. A physical exam revealed tenderness and rebound tenderness on the whole abdomen; hence, we conducted follow-up abdominal CT. Compared to the previous CT scan, mucosal wall thickening along the large bowel and small bowel was newly observed; therefore, we assessed it as enterocolitis, which is highly indicative of immune checkpoint inhibitor (ICI) colitis. After conservative treatment for 1 week, including intravenous hydration and antibiotics, his symptoms were relieved. The patient had minimal bloody stool, but negligible.

However, after 1 week, his symptoms suddenly exacerbated: abdominal pain, diarrhea and hematochezia started. Laboratory findings at this point were as follows: white blood cell count of 4.03 × 10^3^/μL, C reactive protein of 10.21 mg/dL and procalcitonin of 1.2 ng/mL. Stool culture, stool viral and bacterial polymerase chain reaction (PCR), including cytomegalovirus and clostridium difficile, were all negative.

**Figure 3:**
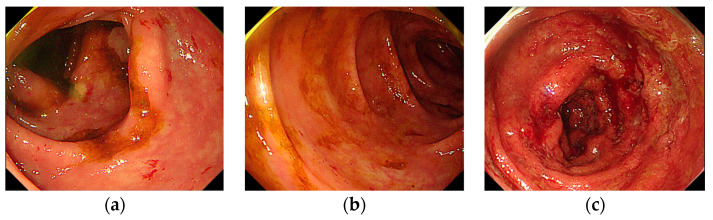
Sigmoidoscopy at the diagnosis of UC mimicking colitis. (**a**) Descending colon. (**b**) Sigmoid colon. (**c**) Rectum.

Thus, we decided to conduct sigmoidoscopy. Sigmoidoscopy revealed continuous and circumferential ulcer and mucosal erythema, blurred vascular marking, friability, mucopurulent exudate, along rectum, sigmoid colon and descending colon, which are typical findings of UC (Figure 3). Mucosal biopsy was carried out, and there was chronic inflammation and crypt architectural distortion, which is also consistent with UC.

**Figure 4:**
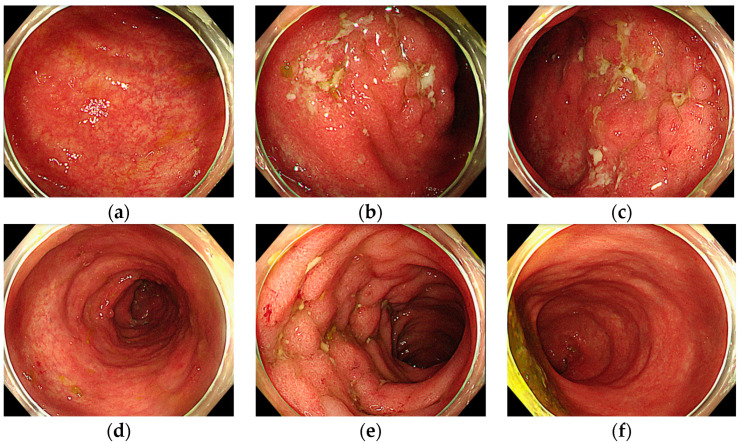
Colonoscopy after treatment of ulcerative colitis: (**a**) ascending colon; (**b**) transverse colon-1; (**c**) transverse colon-2; (**d**) descending colon; (**e**) sigmoid colon; (**f**) rectum.

After sigmoidoscopy, we started oral mesalamine at a dose of 2400 mg twice a day and prednisolone at a dose of 1 mg/kg once a day (or intravenous methylprednisolone equivalently when oral administration was intolerable), considering severe UC with a Mayo score of 3. After 1 week of steroid treatment, his symptoms were relieved. The dosage of prednisolone was decreased to 0.5 mg/kg, while the dosage of mesalamine was kept at the same level. Also, we performed follow-up colonoscopy, and decreases in the extent and intensity of the inflammation was observed (Figure 4). The prednisolone dose was decreased to 30 mg, and to 15 mg subsequently. The improvement in UC allowed us to resume atezolizumab/bevacizumab treatment while maintaining UC treatment with steroids or mesalamine. The patient was discharged after a third cycle chemotherapy as scheduled, maintaining UC treatment with a 7.5 mg dosage of prednisolone daily and 2400 mg of mesalamine twice a day. So far, the patient has been continuing atezolizumab/bevacizumab treatment without reporting any significant gastrointestinal symptoms.

**Figure 5:**
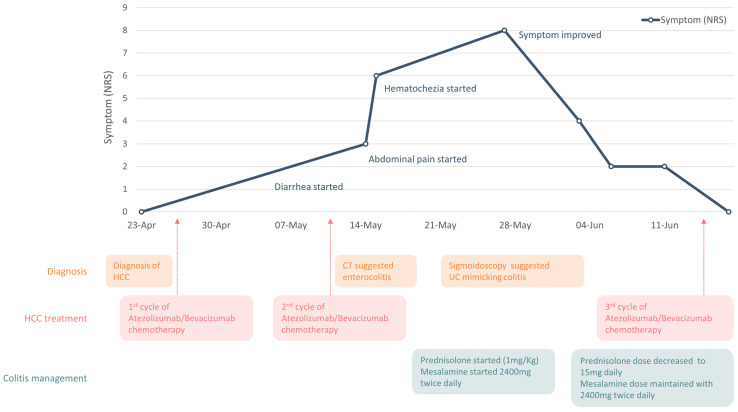
Clinical course and management of patient.

The clinical course and management of the patient is graphically summarized in Figure 5.

## 3. Discussion

ICIs have emerged as pivotal treatments for a variety of cancers, including HCC. Among the adverse effects of ICIs, ICI colitis is a particularly frequent and challenging complication, with reported incidence rates ranging from 1% to 25% depending on the specific ICI agent used. However, there are only a few case reports regarding colitis and atezolizumab, which are summarized in Table 1. And there are even fewer reported cases of UC-mimicking colitis related with atezolizumab. This case report holds great significance as it documents the first instance of UC-mimicking colitis induced by atezolizumab, which responded well to treatment with steroids and mesalamine.

The IMBRAVE 150 trial, which established the atezolizumab/bevacizumab combination as a standard therapy for HCC, reported a 1.8% incidence of colitis, which is three times higher than the 0.6% incidence observed with sorafenib [3]. The symptoms of ICI colitis vary widely, with diarrhea (92%) being the most common, followed by abdominal pain (82%) and hematochezia (64%). Severe cases can lead to complications requiring surgical intervention [17].

Notably, ICIs targeting cytotoxic T-lymphocyte associated protein-4 (CTLA-4) are more likely to cause colitis compared to those targeting programmed cell death protein-1 (PD-1) and programmed cell death-ligand 1 (PD-L1). A histopathological examination of ICI colitis often reveals heavy infiltration of CD8+ T cells, a finding also seen in UC, along with dense lymphoplasmacytic lamina propria expansion, increased intraepithelial lymphocytes and cryptitis [18,19].

Diagnosis of ICI colitis primarily involves the exclusion of other potential causes such as infectious colitis, ischemic colitis, and inflammatory bowel disease (IBD). Laboratory tests often show elevated inflammatory markers like CRP and fecal calprotectin [20]. An early endoscopic evaluation with biopsies is recommended for patients presenting with grade 2 to 4 colitis, with findings such as exudates, the loss of vascular pattern, granular mucosa and erythema, which closely resembles UC [21,22,23]. The biopsies typically show chronic inflammation and crypt architectural distortion, which are consistent with UC [21].

ICI colitis resembles UC in other aspects as well. Interferon gamma and tumor necrosis factor (TNF) alpha play central roles in the pathogenesis of both ICI colitis and UC [24]. Resulting from their shared pathogenesis and endoscopic findings, systemic steroids and biologics are commonly used to manage both diseases.

For grade 1 ICI colitis, symptomatic treatment with hydration and antidiarrheals is generally sufficient. However, grade 2 or higher colitis often requires withholding ICI therapy and administering systemic steroids at doses of 0.5–2 mg/kg. Steroid tapering is crucial to mitigate potential side effects and maintain the anti-tumor efficacy of ICIs [25].

In cases where steroids are ineffective, biologics such as infliximab, vedolizumab and ustekinumab may be used. Infliximab at a dose of 5 mg/kg and vedolizumab at 300 mg typically resolve symptoms more quickly than steroids alone [26,27,28]. There are some case reports regarding ustekinumab treatment, but more large-scale studies must be conducted [29,30,31]. There are also reports of fecal microbiota transplantation being effective in treating refractory ICI colitis [32].

A meta-analysis indicated that 41% of grade 3 ICI colitis cases are steroid-responsive, while all grade 4 cases are steroid-refractory [33]. Higher endoscopic grading scores, such as the Mayo score, often correlate with refractoriness to steroid treatment while not compatible with high symptomatic grade [26]. This underscores the importance of early and accurate endoscopic diagnosis, followed by appropriate treatment tailored to the severity of the colitis.

It is also important to consider the management of ICI colitis in the context of resuming cancer therapy. ICIs are typically resumed after the resolution of grade 2 colitis but are permanently withheld in cases of grade 4 colitis. Decisions about resuming ICI therapy after grade 3 colitis should be individualized based on the patient’s overall response and recovery. A total of 34% of patients who resumed ICI after ICI colitis recurred, and the median time for the recurrence of ICI colitis was 53 days in a retrospective study [34].

This patient presented symptoms that were significantly similar to those of ulcerative colitis (UC). The inflammation markers were elevated, and although it was not initially checked, fecal calprotectin was also elevated at 911.81 mg/kg. The endoscopic findings were consistent with those of UC. Based on other case reports of UC-mimicking colitis induced by nivolumab, treatment with systemic steroids and oral mesalamine was initiated and was proved effective. However, a distinguishing factor is that ICI colitis responds more rapidly and favorably to biologics compared to UC.

## 4. Conclusions

The similarities between ICI colitis and UC suggest that treatment protocols for UC may be effective in managing cases with ICI colitis showing UC mimicking features. In all cases reporting UC mimicking colitis, steroids and 5-ASA were administered and, in some cases, biologic agents used for UC were effective. Additionally, patients with a history of IBD have a higher incidence of developing ICI colitis and frequent IBD flare-ups. Therefore, incorporating lower endoscopy during standard upper endoscopy for variceal screening could help identify underlying IBD, thereby enabling more accurate risk assessment and management of immunotherapy-related complications.

## Figures and Tables

**Table 1:** Literature review on colitis related to atezolizumab in cancer patients treated with ICI.

Year	Country	Sex/Age	Type of Cancer	Onset	Treatment	Prognosis	Discontinuation of Immunotherapy	Ref.
2022	Japan	M/89	HCC	After sixth administration of atezolizumab/bevacizumab: 1200 mg, 15 mg/kg	Systemic steroid (prednisolone)	Completely recovered	Resumed chemotherapy	Fuji et al. [15]
2021	Italy	F/57	NSCLC (adenocarcinoma)	Two weeks after start of atezolizumab monotherapy, dose unknown	Systemic steroid (methylprednisolone and prednisolone)	Completely recovered	Held chemotherapy	Gallo et al. [16]
2022	USA	F/59	SCLC	Four weeks after start of atezolizumab monotherapy, dose unknown	Total colectomy and end-ileostomy	Refractory to systemic steroid and biologics (infliximab, vedolizumab)	Undescribed	Steiger et al. [12] * previous uncontrolled UC
2024	South Korea	M/54	HCC	After second administration of atezolizumab/bevacizumab: 1200 mg, 15 mg/kg	Systemic steroid (methylprednisolone and prednisolone)	Completely recovered	Resumed chemotherapy	The case reported in this study

Abbreviations: M, male; F, female; HCC, hepatocellular carcinoma; NSCLC, non-small-cell lung carcinoma; SCLC, small-cell lung carcinoma.

## Data Availability

Data are contained within the article.
